# Differentiating Trypanosoma cruzi in a Host Mammalian Cell Imaged in Aqueous Liquid by Atmospheric Scanning Electron Microscopy

**DOI:** 10.1128/spectrum.01413-21

**Published:** 2022-01-05

**Authors:** Yuko Takagi, Mari Sato, Masami Naya, Chikara Sato

**Affiliations:** a Biomedical Research Institute, National Institute of Advanced Industrial Science and Technology (AIST), Tsukuba, Ibaraki, Japan; b Health and Medical Research Institute, National Institute of Advanced Industrial Science and Technology (AIST), Tsukuba, Ibaraki, Japan; University of Arkansas for Medical Sciences

**Keywords:** kinetoplastida, electron microscopy, fluorescent image analysis, infectious disease, intracellular parasites, protozoa

## Abstract

Atmospheric Scanning Electron Microscopy (ASEM) is a powerful tool to observe a wet specimen at high resolution under atmospheric pressure. Here, we visualized a protozoan parasite Trypanosoma cruzi over the course of its infection cycle in the host mammalian cell. This is the first observation of intracellular parasite using a liquid-phase EM. Unlike regular SEM, aldehyde-fixed cell body of T. cruzi appears translucent, allowing the visualization of internal structures such as kinetoplast of trypomastigote and nucleus of amastigote. Plasma membrane of the host mammalian cell also appears translucent, which enabled direct observation of differentiating intracellular parasites and dynamic change of host cellular structures in their near-natural states. Various water-rich structures including micro- and macro- vesicles were visualized around T. cruzi. In addition, Correlative Light and Electron Microscopy exploiting open sample dish of ASEM allowed identification of parasite nucleus and transfected fluorescence-labeled parasites soon after internalization, while location of this morphological intermediate was otherwise obscure. Successful visualization of the differentiation of T. cruzi within the host cell demonstrated here opens up the possibility of using ASEM for observation of variety of intracellular parasites.

**IMPORTANCE** Using Atmospheric Scanning Electron Microscopy (ASEM), we visualized interaction between infectious stage of Trypanosoma cruzi and completely intact host mammalian cell. Plasma membrane appears translucent under ASEM, which not only enables direct observation of T. cruzi within its host cell, but also reveals internal structures of the parasite itself. Sample deformation is minimal, since the specimen remains hydrated under atmospheric pressure at all times. This nature of ASEM, along with the open structure of ASEM sample dish, is suited for correlative light-electron microscopy, which can further be exploited in identification of fluorescent protein in the intracellular parasites.

## INTRODUCTION

Infectious diseases caused by eukaryotic intracellular parasites, such as malaria, toxoplasmosis, leishmaniasis, and Chagas disease, impose significant health burden worldwide. Protozoan parasites that cause above diseases go through dynamic morphological and metabolic changes during their life cycles to adapt not only to their vector organisms, but also to intracellular and extracellular environments of their mammalian hosts. Observation of morphological changes of pathogenic parasites within their host cell is of great interest to gain insight into the host-parasite interaction, since it is the most important stage of parasites’ life cycle in drug development research.

Trypanosoma cruzi is a causative agent of Chagas disease, which is transmitted by Triatomine bugs. T. cruzi is excreted by the insect vector as infectious metacyclic trypomastigote. Trypomastigote invades the host mammalian cell by utilizing cellular pathways including phagocytosis, micropinocytosis, and Clathrin-dependent endocytosis (reviewed in Ref. 1). The parasite then encloses itself in parasitophorous vacuole (PV), using host cell endosome and lysosome as membrane resources. Lysosome-mediated vacuole acidification facilitates the parasite differentiation and promotes pore formation by perforin-like proteins Tc-TOX and LYT1, which releases a parasite into the host cytoplasm. The parasite multiplies in the host cytoplasm as amastigote until it exhausts the metabolic resources and then differentiates into trypomastigote to egress from the host cell.

Ultrastructure of T. cruzi has been analyzed by using EM for many years since its first report in 1954 (2). Conventional SEM is utilized extensively to study morphology of free-swimming T. cruzi and those attached to the surface of the host cell. However, presence of host plasma membrane prevents the observation of the parasite during their intracellular infection stage, unless the host membrane is physically removed by dry fracturing method (3) or by membrane extraction (4, 5). On the other hand, TEM has an advantage in investigating detailed subcellular structures, but acquired images are not very intuitive due to the two dimensionality of ultra-thin-sliced specimen. In recent years, 3D reconstruction of serial thin-section TEM images and FIB-SEM tomography overcame those disadvantages, yielding high resolution 3D visuals of intracellular T. cruzi (6, 7). Few drawbacks of these techniques are that image acquisition and processing can be time-consuming and that original sample is unrecoverable due to sectioning or beam milling.

Active T. cruzi is water-rich in nature, including the vesicles secreted by the parasite itself and surrounding PV in the host cytoplasm. Aforementioned EM methodologies require samples to be dehydrated and vacuumed, which may alter original texture and structures of the water-rich host cytoplasmic environment and related membranous and vesicular systems. To overcome this issue, several techniques have been developed recently. Environmental SEM with differential pumping and gaseous electron detector allows imaging of wet samples under low-pressure atmosphere of approximately 1000 Pa (1/100 atm) (8, 9). This technique permits the presence of very thin layer of water above the specimen in a low vacuum at temperatures just above freezing point. For more stable liquid-phase observation, environmental capsules have been developed for TEM (10–12) and SEM (13), which have been successfully used to image hydrophilic molecular complexes (14) as well as cells (15) and tissues (16, 17). Previously, related protozoan parasite T. brucei was observed under SEM in wet condition using such sealed capsule (13). However, intracellular parasites including T. cruzi has never been examined at EM resolution in entirely immersed condition yet.

In this report, our developed Atmospheric Scanning Electron Microscope (ASEM) was used to observe the interaction of T. cruzi and host mammalian cell in aqueous buffer. Open structure of ASEM dish gives considerable flexibility to the experimental protocol. Samples can be analyzed as a whole mount, eliminating the need for sectioning, and enabling re-staining and repeated observation (Fig. S1). ASEM is a powerful tool to allow high resolution observation of the sample immersed in aqueous solution at atmospheric pressure. This report is the first visualization of intracellular protozoan parasite at EM resolution within the intact host cell.

## RESULTS

### Characteristics of ASEM images.

**FIG 1:**
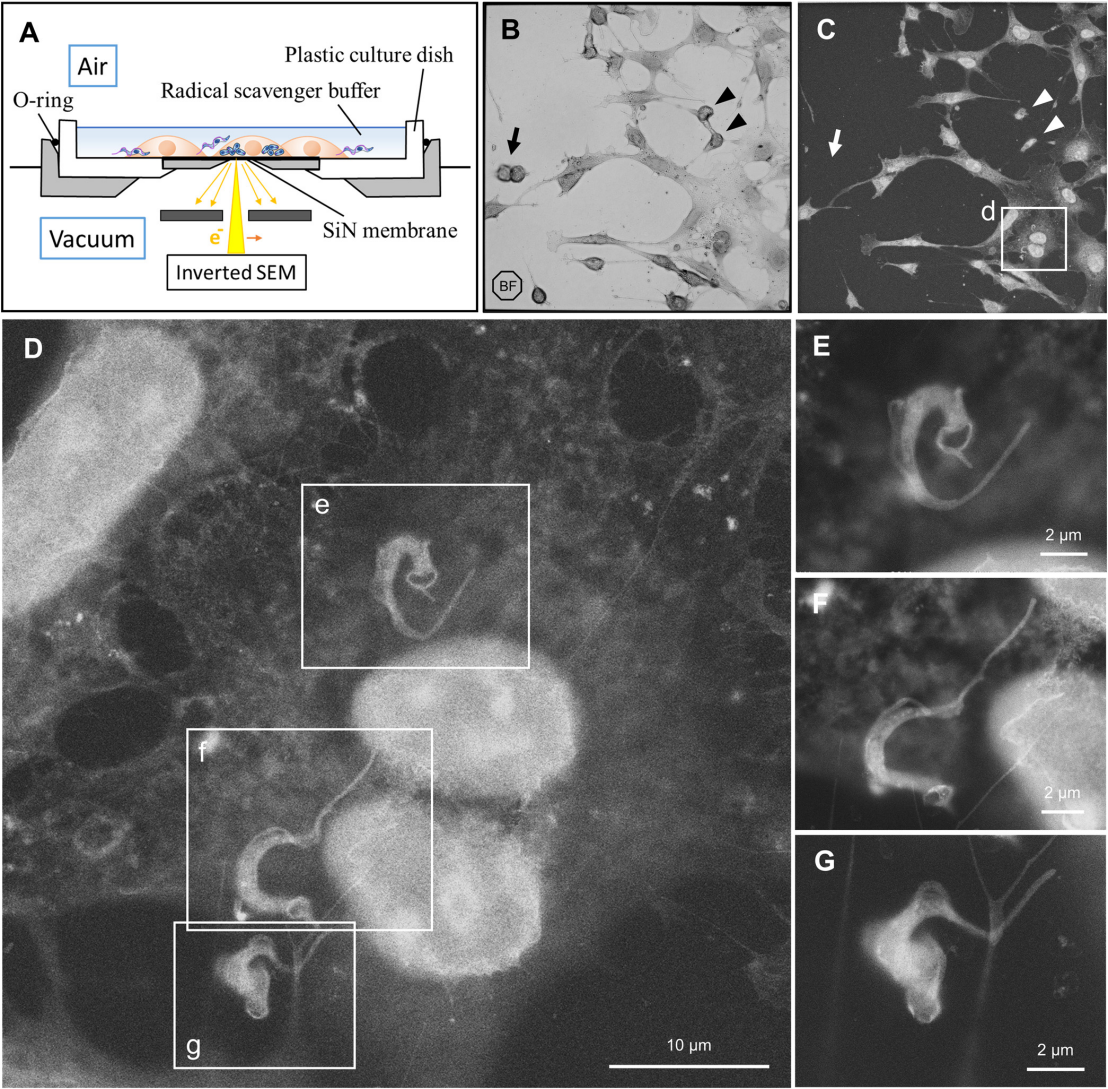
Use of ASEM to visualize the interaction between tissue-derived trypomastigotes and host 3T3 cells. (A) Schematic representation of ASEM set up. Electron beam passes through SiN film window, and backscattered electrons are captured by the electron detector. Biological specimen in aqueous buffer remains in open air. Samples and equipment components are not to scale. (B) Bright field image of a silicon nitride film window of ASEM dish, captured by light microscopy. (C) The same window as (B), observed under ASEM. Three-dimensionality of round 3T3 cell is not entirely reflected in the ASEM image, since the observable specimen depth is smaller than the height of those cells (arrowheads). Some loosely attached 3T3 cells were lost during handling (arrows). (D) Higher magnification of the area (d) in panel (C). (E) Higher magnification of the area (e) in panel (D). (F) Higher magnification of the area (f) in panel (D). (G) Higher magnification of the area (g) in panel (D).

Host 3T3 cells were directly inoculated and cultured overnight on an ASEM dish. Trypomastigote stage of T. cruzi was allowed to interact with the host cells for 4 h before fixation. The SiN film window is transparent and thin, which allows observation of the sample by regular inverted light microscopy (LM) as well as by ASEM (Fig. 1A). Figure 1B and C show the coculture on the identical dish window; the former was imaged by LM and the latter by ASEM. Compared with the bright field image by LM, nuclei of 3T3 cells are clearly visible under ASEM. On the other hand, three-dimensionality of round 3T3 cell is not entirely reflected in the ASEM image, since the observable specimen depth of approximately 2 µm from the SiN membrane at acceleration voltage of 20 kV (18) is clearly smaller than usual height of the 3T3 cells (Fig. 1B and C, arrowheads). Some loosely attached 3T3 cells were apparently lost during handling (arrows).

### Interaction of tissue-derived trypomastigote with host cell.

Several parasites were captured interacting with a host 3T3 cell using ASEM (Fig. 1D). Trypomastigotes in Fig. 1E and F apparently crawled under the host cell because parasites attached on the top surface of the host cell would have been “blurred” due to defocus with this microscopy setting. The trypomastigote in Fig. 1G is interacting with the host cell only through a thread of the cell membrane. Flagellum attachment zone is somewhat loose and translucent, giving undulating membrane-like appearance. This quality is distinct from images captured by conventional SEM in combination with surface metal coating. Also, kinetoplast often appears as a dark spot (Fig. 1E and F). This visual is unexpected, since nucleoprotein-rich regions such as nucleus and mitochondria tend to look bright under ASEM, as in the case with host 3T3 cell, since phosphotungstic acid (PTA) preferentially stains chromatin and proteins (19, 20). The fact that whole cell body of T. cruzi tends to look bright suggests relatively abundant nucleic acids and proteins, potentially reflecting high translation activity in the cytoplasm of the parasite.

**FIG 2:**
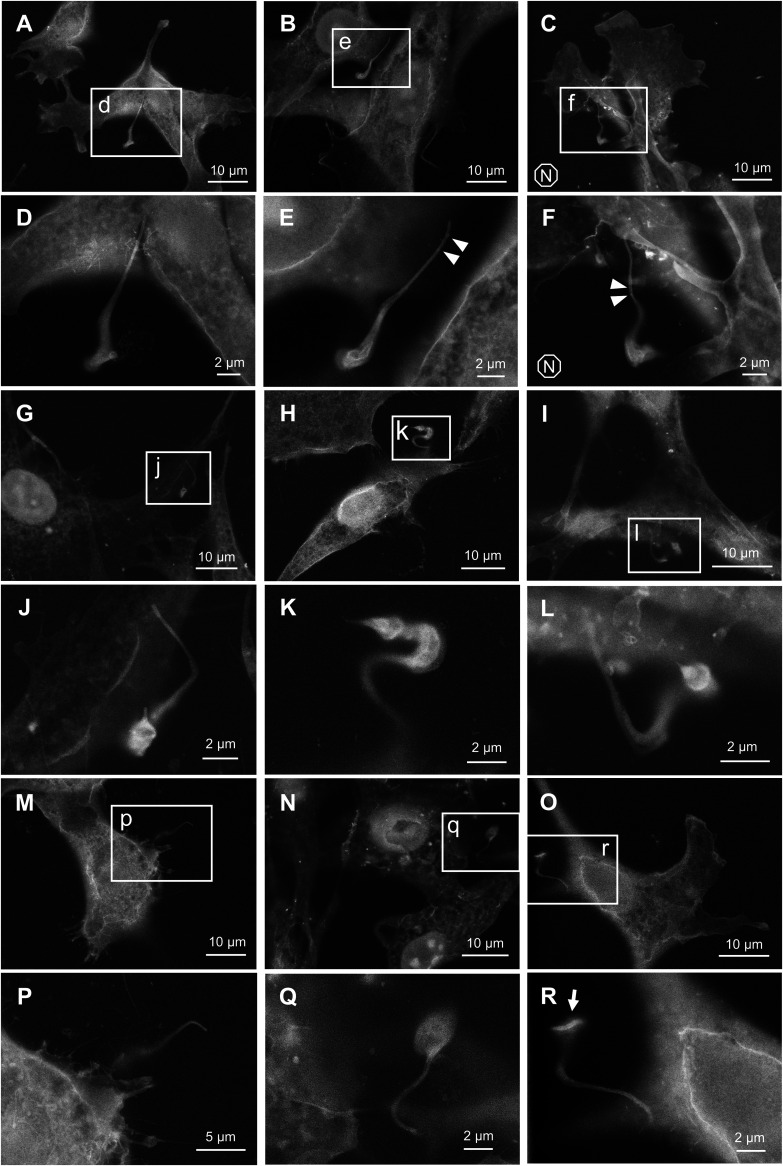
Morphological variation of trypomastigotes upon interaction with host cell. (A–C), (G–I) and (M–O) are ASEM images at lower magnification. (D–F), (J–L), and (P–R) show higher magnification images of subareas indicated by corresponding lowercase letters. Letter N in octagon indicates the sample was re-stained by NCMIR method (C and F). Some speckles were found on flagella surface of trypomastigotes, which can potentially be extracellular vesicles (E and F, arrowheads).

Trypomastigotes take various shapes upon interaction with host cells (Fig. 2). In most cases, not only the outlines but also the internal structures of parasite cells were visible. Many trypomastigotes were found invading the host cell by their anterior ends (Fig. 2D–F, K and R). Straight, stiff-looking flagella were often observed in those parasites (Fig. 2D and E). On the other hand, some parasites were attached to the host cell by their posterior ends (Fig. 2P and Q). This is consistent with a previous report that trypomastigotes can invade the host cell by either direction (21). In some rare cases, parasite’s cell body seemed to be adhered to the host cell more tightly than its flagella or posterior end (Fig. 2J and L). Position of kinetoplast was easily identifiable in many trypomastigotes, either by the difference in electron density or by its lumpy appearance (Fig. 2F and J–L). However, in other cases, position of kinetoplast is obscure (Fig. 2D, E, Q, and R). In addition, some trypomastigotes showed distinct ridge-like structure (Fig. 2R, arrow), potentially representing a sharply curved flagellum, associated with twisted cell body (22, 23). We also observed tadpole-shaped parasites (Fig. 2Q). This could be a transition stage between trypomastigote and amastigote, but also can be a spheromastigote, which is a variant closer to epimastigote.

**FIG 3:**
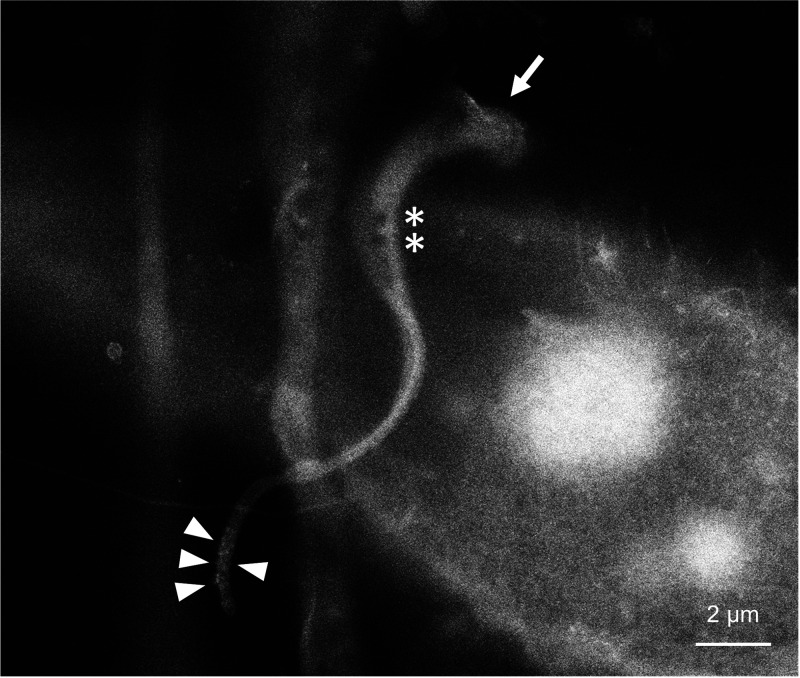
Invading trypomastigote. Spiral grooves at the entry site resemble coiling phagocytosis (arrow). Vacuole-like structures in parasite cell body are visible (asterisks). Tip of flagella is covered by speckles, which appear to be extracellular vesicles (arrowheads).

Some parasites are entangled with filopodia-like membrane extension of the host cell (Fig. 1F, 1G, 2P, and Fig. 3). This membrane projection was especially prominent in Fig. 2P. Similar host-parasite interaction was previously observed in HeLa cell, in which plasmalemma projection subsequently covered invading trypomastigote as a membrane “sleeve” (24). We also observed a parasite with distinct grooves at its host entry site (Fig. 3, arrow). This structure is likely to be a phagocytotic cup of the host plasma membrane, which is within the observable specimen depth within almost 2 μm from the SiN film. Due to the high resemblance to the SEM image reported by Barrias et al. (21), it is tempting to speculate that this spiral groove represents an event of coiling phagocytosis, which is a rare internalization pathway for T. cruzi (25).

Surface of trypomastigote occasionally has small bumps especially on flagella (Fig. 2E and F and Fig. 3, arrowheads), which can potentially be extracellular vesicles, as the size of particles is similar to the ones in previously reported SEM images (26, 27). In addition, vacuole-like dark spots were observed in the parasite cell body (Fig. 3, asterisks), suggesting that water-rich subcellular structures can be visualized by ASEM.

With these results, we applied ASEM to various developmental stages of T. cruzi.

### Host cell invasion and morphological transition.

**FIG 4:**
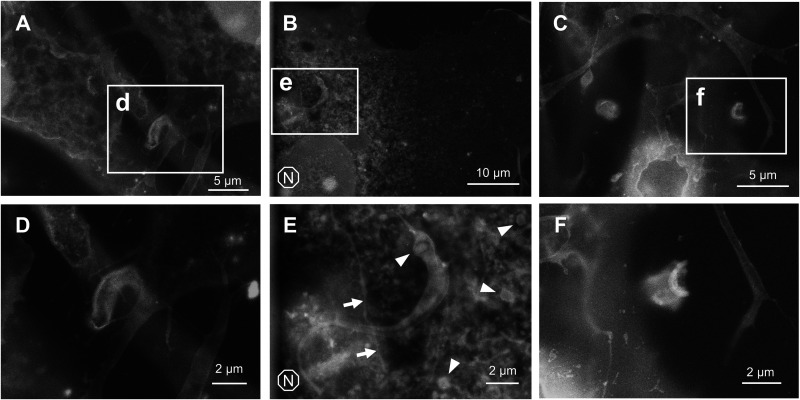
Invasion of host cell by trypomastigotes (A) trypomastigote partially covered by host plasma membrane. (B) Trypomastigote in host cytoplasm. (C) Curled trypomastigote interacting with the host plasma membrane. (D) Higher magnification of area (d) in panel (A). (E) Higher magnification of area (e) in panel (B). Host cell fiber (arrows) and granule-like structures (arrowheads) overlap with trypomastigote, indicating the parasite is inside the host cytoplasm. (F) Higher magnification of area (f) in panel (C).

Internalization of T. cruzi, including PV formation and cytoplasmic escape, can be completed as early as 2-h postinfection (6, 28). In our samples with 3T3 cells at 4-h postinfection, we found some parasites that appear to be in the middle of internalization process (Fig. 4). Figure 4D shows a trypomastigote beginning to be covered by the host cell membrane, and Fig. 4F shows a trypomastigote curled up upon entry into the host cell. In Fig. 4E, the parasite is at least partly inside the host cell, although the morphology of the parasite resembles ordinally trypomastigote. The fact that host cytoplasmic fiber runs across flagellum (arrows) and a cytoplasmic granule overlaps with the parasite cell body (arrowheads) together indicate that the trypomastigote is located within the cytoplasm, not just crawled beneath the host cell.

**FIG 5:**
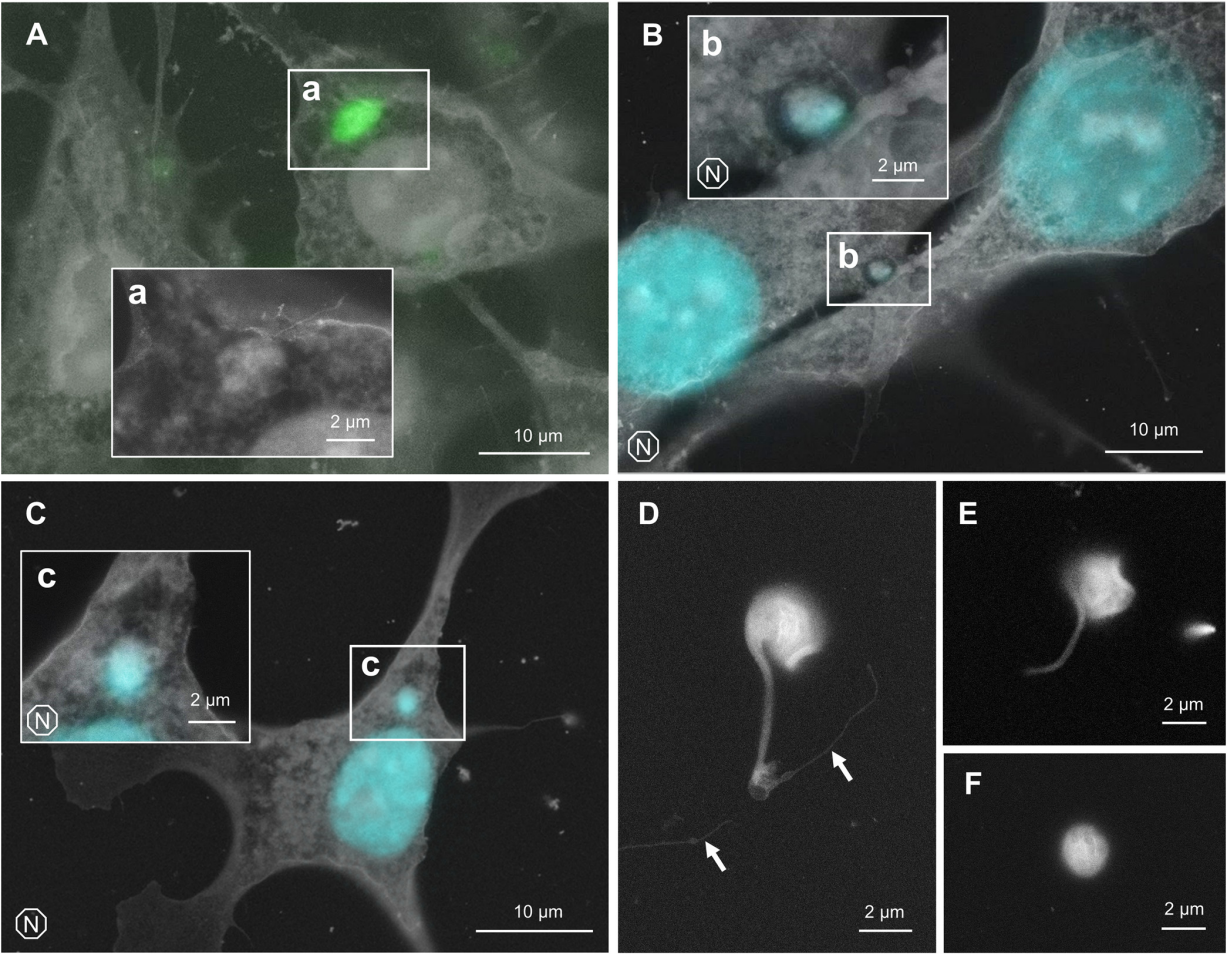
Differentiation of trypomastigote to amastigote. (A) GFP-expressing T. cruzi was used to locate the recently-internalized parasite. Overlay of ASEM and fluorescence microscopy images is shown. Higher magnification of subarea (a) is shown without overlay. (B) Hoechst staining was performed to distinguish T. cruzi from host organelles. Overlay of ASEM and fluorescence microscopy images is shown. (C) Hoechst staining was performed to locate recently-internalized parasite. Overlay of ASEM and fluorescence microscopy images is shown. (D) Differentiating trypomastigote outside of host cell. Threads of host plasma membrane are indicated by arrows. (E) Intermediate form between trypomastigote and amastigote, found outside the host cell. (F) Extracellular amastigote.

Some parasites are completely internalized and already transformed into round form at 4-h postinfection (Fig. 5). It is difficult to locate a differentiating parasite soon after internalization, possibly due to the presence of PV membrane or due to the distance to the SiN film of ASEM dish. This temporary “disappearance” coincides with the time window when a parasite becomes indistinguishable from the host cytoplasm under a phase-contrast microscope (29). To distinguish the parasite from host organelles and other intracellular structures, GFP-expressing T. cruzi was employed to identify the location of internalized parasite (Fig. 5A). Alternatively, nuclear staining was performed prior to ASEM imaging, and the fluorescence microscopy image and ASEM image were overlaid to identify the location of DNA of the parasite (Fig. 5B and C). In Fig. 5A and C, it was difficult to delineate exact shape of differentiating T. cruzi. In Fig. 5B, amastigote is situated in a hollow cavity, which may represent a phagocytotic cup often observed when extracellular amastigote is internalized (30), although it is not clear whether this particular amastigote was already differentiated before internalization or not.

We did not come across with any intracellular parasite that is definitively in a transition stage between trypomastigote and amastigote, mainly because of the ambiguity of the parasite cellular boundary soon after host invasion. Meanwhile, trypomastigote is known to differentiate into amastigote without host cell, if kept long in a culture medium (31). Indeed, we observed some extracellular parasites that are going through *in vitro* amastigogenesis (Fig. 5D and E, and 5F). Based on the appearance of those parasites, cell body seems to be twisted and curled as flagellar starts shortening. Similar morphological change can be found in previously reported SEM images (32). Some parasites were found attached to a fragment of putative cell philopodium (Fig. 5D, arrows), which might have promoted the differentiation (33).

### Intracellular replication.

**FIG 6:**
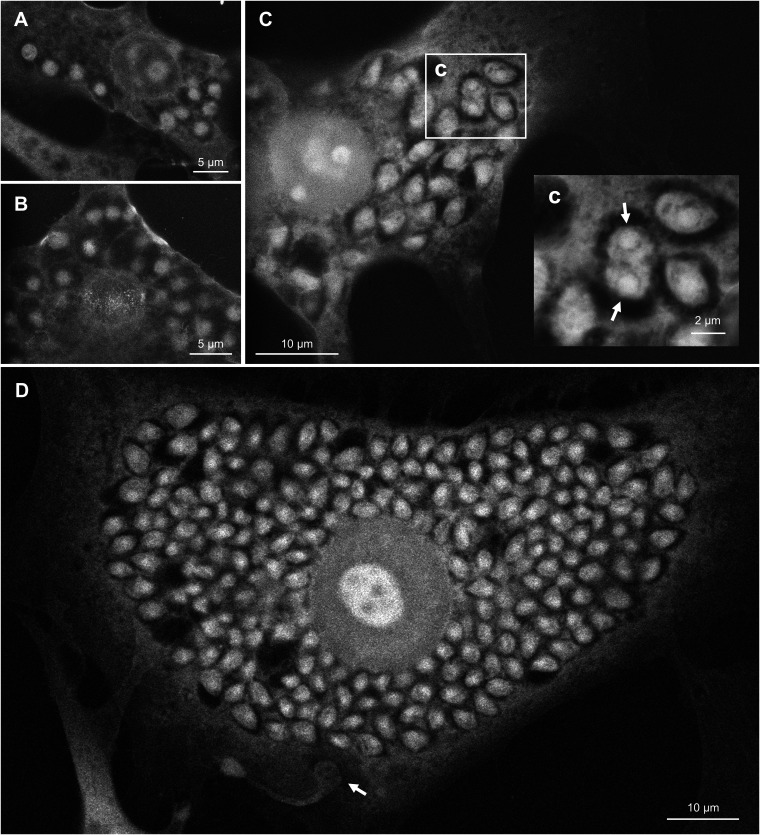
Intracellular replication of amastigote. (A) Host 3T3 cell harboring amastigotes, 2 days postinfection. (B) Host 3T3 cell harboring amastigotes, 3 days postinfection. (C) Dividing amastigote in host 3T3 cell. Higher magnification of area (c) is shown. Nuclei of dividing cell are indicated by arrows. (D) Hose 3T3 cell filled with amastigotes. Potential intermediate form of parasite is indicated by an arrow.

Once amastigotes are fully differentiated, their appearance become much more prominent (Fig. 6). Amastigote looks more distinct, partly because it displaces the host cytoplasmic contents to form a compartment-like space around itself. From 2 to 3 days postinfection, the shape of amastigote appears to be close to a sphere, similar to the extracellular amastigote in Fig. 5F (Fig. 6A and B). However, in later time periods, it becomes more oval and somewhat rhombic or triangular (Fig. 6C and D). Position of nucleus also becomes easier to identify. Occasionally, we observed amastigotes in the middle of binary fission (Fig. 6C, arrows). Timing of the cell division is not synchronous with neighboring amastigotes, which is consistent with previous reports (34). In Fig. 6D, we found a parasite in an unusual form (arrow). This can be a transition intermediate from amastigote to trypomastigote, trying to escape from the host cell. Alternatively, it can be a trypomastigote trying to sneak into an already-infected host cell. In the latter case, it can be a similar morphological variation to the parasite in Fig. 2Q.

### Differentiation from amastigote to trypomastigote.

**FIG 7:**
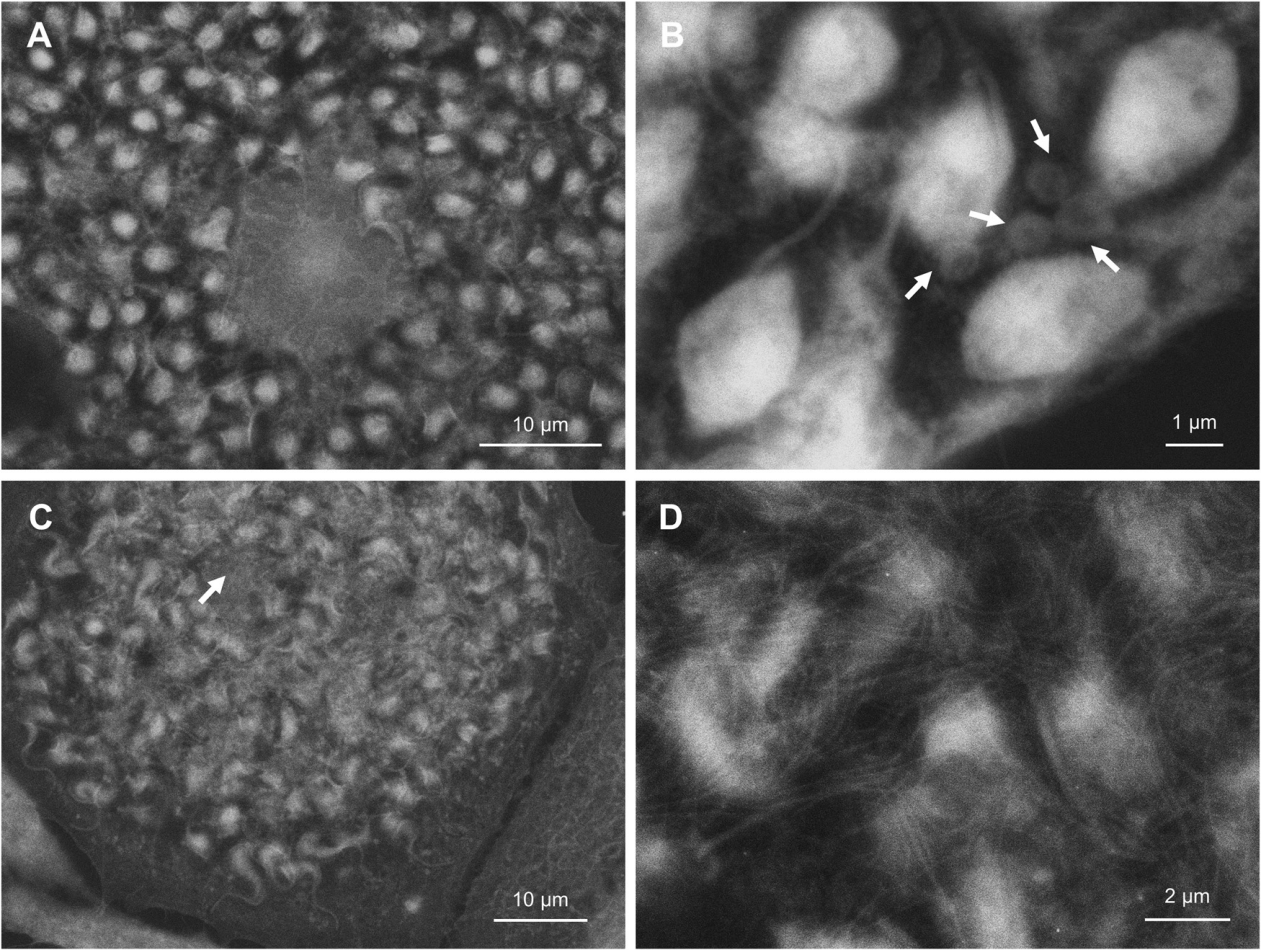
Differentiation of amastigote into trypomastigote. (A) Intracellular parasites, 4 days postinfection. Flagella is extended while cell body is still round. (B) High resolution image of parasites in similar infection stage to (A). Vacuole-like host cytoplasmic structures are indicated by arrows. (C) Host cell with disintegrated nucleus, 5 days postinfection. Remnant of host nucleus is indicated by an arrow. Shape of parasite cell body and its flexibility suggests that differentiation to trypomastigote is completed. (D) High resolution image of parasites in similar infection stage to (C), entangled with fibers.

By 4 days postinfection, some amastigotes have started differentiating into trypomastigotes (Fig. 7A). Host cytoplasm is filled with parasites, and the host nucleus has started to disintegrate. Amastigotes already have considerably long flagella, even though many of their cell bodies remain oval (Fig. 7B). Host cytoplasm is going through a dynamic change at this point. Vacuole-like structures are observed together with parasites (Fig. 7B, arrows), which are less prominent in early phase of infection. By 5 days postinfection, parasite cell body is elongated and largely resembles that of trypomastigote (Fig. 7C). Host cell nucleus is totally disintegrated and almost indistinguishable in many cases (arrow). String-like structures of unknown origin is occasionally seen in this phase (Fig. 7D). This fiber could potentially be a host chromatin, but identification of its true nature requires further investigation.

### Egress.

**FIG 8:**
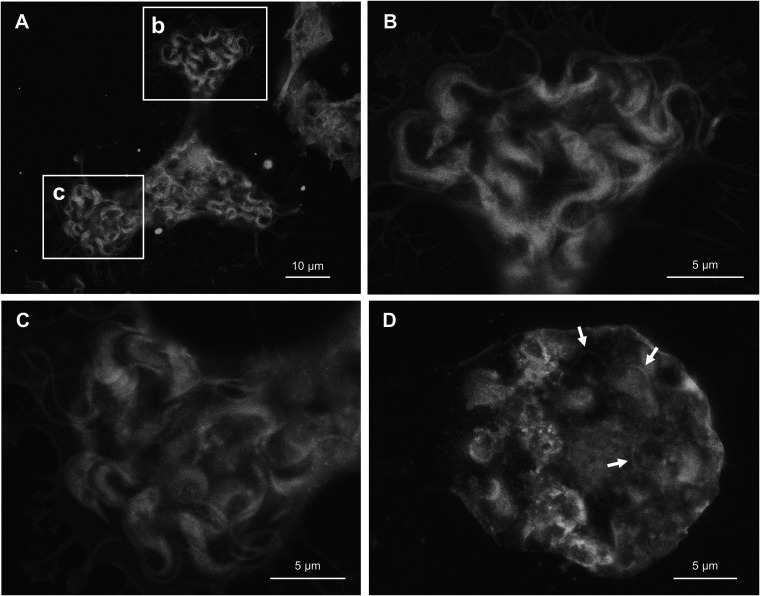
Differentiated trypomastigote and host cell debris after egression. (A) Host 3T3 cell containing fully-differentiated trypomastigotes, 5 days postinfection. Plasma membrane is stretched and peeled due to tension caused by motile parasites. (B) Higher magnification of area (b) in panel (A). (C) Higher magnification of area (c) in panel (A). (D) Host cell debris after trypomastigote egression. Flagella of trapped, under-differentiated parasites are indicated by arrows.

By 5 days postinfection, trypomastigotes start to egress from the host cell. Host cells filled by fully differentiated trypomastigotes are often partially peeled off from the culture dish surface, presumably due to the tension caused by highly motile trypomastigotes (Fig. 8A). Stretched membrane threads spread radially from the host cell body to hold on to the dish surface. Fully differentiated trypomastigotes within the host cell have dark kinetoplast and translucent flagellum attachment zone (Fig. 8B and C), which are consistent with what was observed in Fig. 1 and 2. Once a host cell ruptured and trypomastigotes egressed, the membrane of the host cell was deflated (Fig. 8D). There are some parasites trapped inside of the host cell debris (arrows), probably because of insufficient motility due to incomplete differentiation. It is consistent with previous reports that intracellular parasites do not multiply and differentiate in synchrony (34), and some still remain as amastigote at the time of host cell rupture (5).

## DISCUSSION

We demonstrated for the first time that ASEM is an effective tool to visualize an intracellular protozoan parasite in its near-natural state. Images of T. cruzi captured by ASEM have rather similar taste to traditional LM images, comparing to conventional SEM that gives the surface thick solid texture. Under ASEM, plasma membrane appears translucent, which not only allows to see through the internal structure of T. cruzi itself, but also enables the observation of differentiating intracellular parasites within the host cell without the need to tear open the obstructing plasma membrane of the host cell. Indeed, appearances of extracellular trypomastigotes before invasion and intracellular trypomastigotes prior to egression show similar characteristics, such as dark kinetoplast, bold flagella and faint flagellum attachment zone resembling undulating membrane, suggesting that presence of host plasma membrane does not significantly interfere with the observation (Fig. 1 and 8). Likewise, the image of extracellular amastigote has similar morphological nature to that of intracellular amastigotes in early replication stage before they turn into oval in later time points (Fig. 5F and 6A).

Trypomastigote of T. cruzi is known to have morphological variability, traditionally described as slender, broad, short, very broad and intermediate forms (35). However, our observation indicates that the parasites can take even more diverse forms upon interaction with the host cell. Parasites may initiate the cytoskeletal rearrangement prior to the internalization into the host cell. In fact, previous study showed that phosphorylation state of many T. cruzi proteins changes upon interaction with the extracellular matrix, resulting in the metabolic reprogramming in the invading trypomastigote (33).

Phosphotungstic acid stains positively charged groups of proteins without significantly affecting glycogen or lipid (36). The visual of amastigotes displacing host cytoplasmic components may reflect the activity of proteases associated with the intracellular parasite. Proteases are known pathogenic factor of T. cruzi, some of which are implicated as drug targets (37). Some proteases are expressed throughout all life cycle stages, and others are differentially expressed (38). In amastigote stage, cruzipain 2, an isoform of the major cysteine protease cruzain, is localized on the cell surface (39, 40). It is plausible that cruzipain 2 or other protease digest surrounding host protein components for nutrient acquisition, which might have led to the appearance of hollow compartment around amastigotes. In fact, it has been documented in a closely related kinetoplastid parasite *Leishmania* that amastigotes secrete proteases (41) and possibly utilize digested peptides in PV. Although it is not clear whether amastigote of T. cruzi releases cruzipain or other protease, cruzipain is secreted by trypomastigote (42) and activity of cruzipain is indeed required for intracellular proliferation of amastigote (43). Alternatively, this compartment-like cytoplasmic structure may represent extensive branching of host mitochondrial network. Lentini et al. have demonstrated that intracellular amastigotes maintain close proximity to host mitochondria by attaching the remanent of flagellum to the mitochondrial membrane (44). Our staining method cannot distinguish mitochondria from other subcellular structures, so it is difficult to confirm this notion. However, we occasionally observed amastigotes associated with a host cytoplasmic component by an ambiguous thread (Fig. 6A and B). These fibrous structures might represent the reported connections.

It was difficult to locate T. cruzi soon after host invasion, potentially due to the presence of PV or simply due to the distance to the focal plane. To that end, Correlative Light and Electron Microscopy (CLEM) was employed to confirm the position of intracellular parasites, either by GFP expression or by nuclear staining (Fig. 5). Sample observation window of ASEM dish is made of thin SiN film, which allows the use of light fluorescence microscopy. The biggest advantage of using ASEM in CLEM is that sample deformation is minimal between the two imaging events, since the sample remains under constant hydration. This nature of ASEM allows precise image overlay, suited for variety of subcellular localization analyses. Kurup et al. demonstrated that trypomastigote undergoes asymmetric division to discard flagellum during amastigogenesis (45). CLEM using fluorescent antibody labeling against flagellar protein and ASEM both from top and bottom of the cells can potentially visualize such event as well (46).

Yet another informative aspect of ASEM images in this study is the visualization of dynamic change of the host mammalian cell. In non-infected host 3T3 cells and in early infection phase up to amastigote replication, nucleus of host cell is clearly visible, including densely stained nucleolus. However, by the time amastigote differentiates into trypomastigote, border of nucleus becomes obscure and disintegrates in many cases. Vesicle-like structures often appeared among crowded parasites. Also, we occasionally observed fibers of unknown origin in this stage of host cytoplasm, which could potentially be a spilled chromatin, but further investigation is required to identify its nature.

Interaction between T. cruzi and 3T3 cell also demonstrated the elasticity of plasma membrane. Prior to infection, peripheral structure of 3T3 cell mostly resembles lamellipodia. However, many trypomastigotes appeared to be entangled with philopodia-like strings. In late infection phase close to parasite egression, cell periphery extends narrow membrane protrusions to hold onto the dish surface. Membrane branching at this stage is much more extensive comparing to regular philopodia. Anchoring points on the dish surface may represent the location of focal adhesion site (47), which is likely to linger as cell body is peeled and membrane is regressed due to the tension caused by motile trypomastigotes.

Taken together, this work demonstrated that ASEM is a powerful tool to visualize the interaction of intracellular parasite and its mammalian host cell. Successful observation of T. cruzi opens up the possibility of using ASEM in the study of variety of other intracellular pathogenic protozoans, such as *Leishmania* spp., *Plasmodium* spp., Toxoplasma gondii and Cryptosporidium parvum. With the combination of high-resolution imaging and intuitive visual output, ASEM can potentially uncover new aspect of the protozoan biology.

## MATERIALS AND METHODS

### Parasite culture.

Infectious stage of T. cruzi Tulahuen strain was maintained as a coculture with Swiss 3T3 host cells in DMEM containing 10% FBS at 37°C in a CO_2_ incubator. Wildtype parasite was used for the protocol optimization and time course experiment. Parasite harboring the GFP expression vector pTREX-EGFP (48) was used to visualize the intracellular parasite under fluorescence microscopy. Trypomastigote stage of T. cruzi was harvested from the supernatant of host-parasite coculture by centrifuging the mixture of trypomastigote, amastigote and host cell debris, and letting the active trypomastigote swim out of the pellet (49). To establish a fresh infection, host 3T3 cells were seeded in an 8-window ASEM dish (JEOL Ltd., Japan) (50) 1 day prior to the infection, and trypomastigote was added to the dish in MOI of 10. For the time course experiment, the coculture was kept on the same dish for the first 2 days and was passaged to prepare samples of later time points avoiding culture saturation.

### Microscopy specimen preparation.

The host-parasite coculture was fixed on the ASEM dish by 4% formaldehyde in PBS (pH 7.4) for 20 min at room temperature. GFP fluorescence of the parasites was imaged at this stage. The sample was further fixed with 1% glutaraldehyde solution at room temperature in 0.1 M cacodylate buffer (CB, pH 7.4) for 20 min. The fixative was washed by immersing the sample with 0.1 M CB for 3 min, twice. The sample was kept in 0.5% sodium azide in PBS for storage.

### Staining.

Positively charged 1.4 nm Nanogold particles (PCG; #2022, Nanoprobes, NY, USA) are known to preferably stain flagella of bacteria and plasma membrane of cells (51). After counter fixation of the cells with glutaraldehyde, samples were incubated with a 6 μM solution of PCG on an ASEM dish for 20 min at RT (51). After washing with double distilled water (DDW), particles were enlarged by gold sedimentation using GoldEnhance (#2113, Nanoprobes) to be visualized by ASEM of 8 nm resolution. After washing with DDW, the sample was further incubated with 2% PTA to stain surrounding positively charged proteins and chromatin.

Some samples on the ASEM dishes were counter stained using the NCMIR method (52). Briefly, the fixed cells were further fixed/stained with 0.15 M CB supplemented with 2 mM CaCl_2_, 1.5% potassium ferricyanide (Sigma-Aldrich, St. Louis, MO, USA) and 2% aqueous osmium tetroxide (OsO_4_) (Nisshin EM) at RT for 20 min. After washing with DDW, cells were incubated with filtered 1% thiocarbohydrazide (TCH; Tokyo Chemical Industry, Co., Ltd., Tokyo, Japan) at RT for 20 min, rinsed with DDW, and stained with 2% aqueous OsO_4_ at RT for 30 min. After rinsing with DDW, the cells were stained with 2% UA in DDW and kept at 4°C overnight. Finally, after rinsing with DDW, the samples were stained with 0.4% LC at RT for 2 min. Samples counter stained by NCMIR method are indicated by letter N in octagon in figures.

### ASEM imaging.

ASEM images were captured by using the ClairScope (JASM-6200; JEOL Ltd., Tokyo, Japan) (53). The samples were scanned through 100 nm-thick silicon nitride (SiN) film windows at acceleration voltage of 20 kV (19). The cells were immersed in radical scavenger observation buffer [10 mg/ml (wt/vol) D-glucose (Dextrose; MP Biomedicals LLC, Illkirch, France), 1 mM CB (pH 7.4), and 60 mM KCl] (50), and immediately imaged by the inverted SEM of the ASEM at an acceleration voltage of 20 kV. The electron dose at the highest magnification of 11,000× was 0.76 e^-^/Å^2^, which is 1.6% of the dose of 47 e^-^/Å^2^ permitted in low-dose cryo-electron microscopy aiming at atomic resolution single particle reconstructions.
