# Supplementary material for: Differentiating Trypanosoma cruzi in a Host Mammalian Cell Imaged in Aqueous Liquid by Atmospheric Scanning Electron Microscopy
Source: Microbiol Spectr. 2022 Jan 5;10(1):e01413-21. doi: 10.1128/spectrum.01413-21 (PMC8729778; doi:10.1128/spectrum.01413-21)
Supplement: SUPPLEMENTAL FILE 1 — Supplemental material. Download SPECTRUM01413-21_Supp_1_seq18.pdf, PDF file, 0.5 MB [file spectrum01413-21_supp_1_seq18.pdf]

Supplemental Figure 1

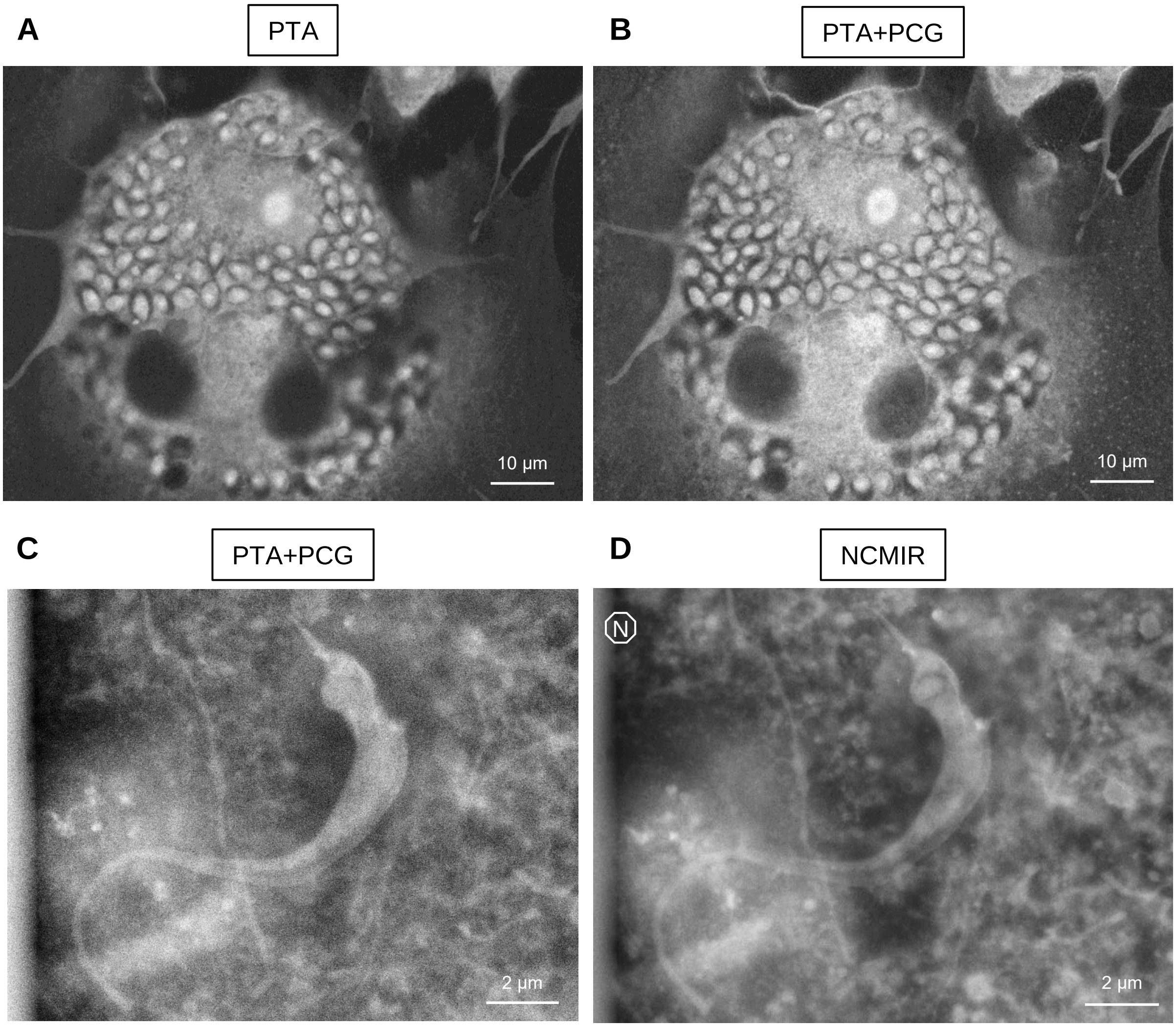

Figure S1. Effect of sequential staining. (**A**) PTA staining of host 3T3 cell harboring amastigotes. (**B**) PTA and PCG staining of the same sample as (A). (**C**) PTA and PCG staining of trypomastigote. (**D**) The same sample as (C), re-stained by NCMIR method.
